# Improving school children’s understanding of water scarcity with a co-produced book on groundwater in Central Chile

**DOI:** 10.1007/s10040-023-02641-6

**Published:** 2023-06-05

**Authors:** Sofía Vargas-Payera, Matías Taucare, Claudio Pareja, Jessica Vejar

**Affiliations:** 1 Centro de Excelencia en Geotermia de Los Andes, CEGA, Departamento de Geología , Facultad de Ciencias Físicas y Matemáticas, Universidad de Chile, Plaza Ercilla 803, 8370450 Santiago, Chile; 2 ETH Zurich, D-USYS TdLab, ETH, Zurich, Switzerland; 3 Departamento de Geología, Facultad de Ciencias Físicas y Matemáticas, Universidad de Chile, Santiago, Chile; 4 Centro Avanzado para Tecnologías del Agua (CAPTA), Facultad de Ciencias Físicas y Matemáticas, Universidad de Chile, Santiago, Chile; 5 Centro de Estudios del Desarrollo Regional y Políticas Públicas (CEDER), Universidad de Los Lagos, Osorno, Chile; 6 Pontificia Universidad Católica de Chile, Santiago, Chile

**Keywords:** Chile, Groundwater and society, Groundwater science communication, Transdisciplinarity, Participatory methods

## Abstract

Water scarcity is a critical issue worldwide, and Chile is no exception. Since 2010, Central Chile has been enduring an ongoing water crisis due to the coupled effects of a severe drought and the overuse of water resources, especially groundwater. Rural communities have been strongly impacted, mainly because wells from which drinking water is supplied show a dramatic drop in water levels, and some have even dried up. The water scarcity scenario requires the integration of actors and disciplines to increase awareness of groundwater; however, how to make this valuable element visible in society is an issue that remains open to debate. This paper describes and reflects on the process of making educational material about groundwater and water scarcity for children to promote public awareness. Based on transdisciplinary and co-designing processes, this work describes the social perceptions of groundwater among children and community leaders, as well as how scientific information and local knowledge of water scarcity could be integrated into a book for the young population. This research finds that educational projects on groundwater resources increase people’s awareness of the role of this hidden resource in the water cycle. Such projects encourage the creation of grounded and contextualised materials that incorporate the knowledge and experience already present in the communities, increasing public awareness of the role of groundwater and associated water scarcity issues, thereby integrating academia and society. This approach could be a tool to lay the foundations for successfully addressing the water crisis in Chile over generations.

**Supplementary Information:**

The online version contains supplementary material available at 10.1007/s10040-023-02641-6.

## Résumé

La rareté de l’eau est un problème crucial dans le monde entier, et le Chili ne fait pas exception à la règle. Depuis 2010, le centre du Chili est confronté à une crise de l’eau permanente en raison des effets conjugués d’une grave sécheresse et de la surexploitation des ressources en eau, en particulier des eaux souterraines. Les communautés rurales ont été fortement impactées, principalement parce que les puits d’approvisionnement en eau potable ont connu une baisse spectaculaire des niveaux d’eau, et certains se sont même asséchés. Le scénario de la pénurie d’eau nécessite l’intégration d’acteurs et de disciplines pour accroître la sensibilisation aux eaux souterraines. Cependant, la question de savoir comment rendre cet élément précieux visible au sein de la société reste ouverte au débat. Cet article décrit et réfléchit au processus de création de matériel éducatif sur les eaux souterraines et la pénurie d’eau pour les enfants afin de promouvoir la sensibilisation du public. Basé sur des processus transdisciplinaires et de co-conception, ce travail décrit les perceptions sociales des eaux souterraines chez les enfants et les dirigeants communautaires, ainsi que la manière dont les informations scientifiques et les connaissances locales sur la pénurie d’eau pourraient être intégrées dans un livre destiné à la population jeune. Cette recherche montre que les projets éducatifs sur les ressources en eaux souterraines sensibilisent les gens au rôle de cette ressource cachée dans le cycle de l’eau. De tels projets encouragent la création de matériaux fondés et contextualisés qui intègrent les connaissances et l’expérience déjà présentes dans les communautés, en sensibilisant le public au rôle des eaux souterraines et aux problèmes de pénurie d’eau associés, intégrant ainsi le milieu universitaire et la société. Cette approche pourrait constituer un outil permettant de mettre en place les bases d’une résolution réussie de la crise de l’eau au Chili sur plusieurs générations.

## Resumen

La escasez de agua es un tema crítico en todo el mundo, y Chile no es una excepción. Desde 2010, Chile Central atraviesa una crisis hídrica debido a factores como una grave sequía y un uso excesivo de los recursos hídricos, especialmente de las aguas subterráneas. Las comunidades rurales se han visto fuertemente afectadas, principalmente porque los pozos de los que se abastecen de agua potable muestran una drástica caída en los niveles de agua y muchos de ellos incluso se han secado. Este escenario de escasez hídrica demanda una integración de actores y disciplinas para aumentar el nivel información y cuidado sobre el agua subterránea. Sin embargo, cómo hacer visible este valioso elemento entre la sociedad es una cuestión que sigue abierta al debate. En ese marco, este artículo describe y reflexiona sobre el proceso de elaboración de material educativo sobre agua subterránea y escasez hídrica para un público infantil, con el fin de promover la concientización pública sobre este vital elemento. Basado en procesos transdisciplinares y de co-diseño, este trabajo describe las percepciones sociales sobre el agua subterránea entre niñas (os) y líderes comunitarios, así como la forma en que la información científica y el conocimiento local sobre la escasez de agua podrían integrarse en un libro. Esta investigación confirma que los proyectos educativos transdiciplinarios aumentan el nivel de consciencia entre la población sobre el rol del agua subterránea en el ciclo del hidrogeológico y expande la mirada de equipos especialistas. Este tipo de proyectos fomenta la creación de materiales educativos situados y con pertinencia local, incorporando el conocimiento y la experiencia ya presente en las comunidades e integrando así el mundo académico y la sociedad. Este enfoque podría ser una herramienta para sentar las bases que permitan abordar la crisis del agua en Chile a lo largo de generaciones.

## Abstract (zh)

全球性的水资源短缺问题日益严峻，智利也不例外。自2010年以来，智利中部一直面临严重的水危机，原因是严重的干旱和水资源(尤其是地下水)过度利用的影响。农村社区受到的影响尤为严重，主要原因是供应饮用水的水井水位急剧下降，有些甚至已经干涸。水资源短缺的情况需要整合各方面的力量和知识，以提高人们对地下水的认识。然而，如何使这个重要的元素在社会中可见仍然是一个待解决的问题。本文描述和反思了制作关于地下水和水资源短缺的教育材料，以促进公众意识的过程。基于跨学科和联合设计的过程，本研究描述了儿童和社区领导人对地下水的社会认知以及如何将科学信息和当地对水资源短缺的认识融入到面向年轻人的书籍中。这项研究发现，地下水资源教育项目可以增加人们对这一隐蔽资源在水循环中的作用的认识。这些项目鼓励创建基于社区已有知识和经验的实地和情境化的材料，提高公众对地下水及相关水资源短缺问题的认识，从而将学术界和社会融合在一起。这种方法可以为几代人解决智利的水危机奠定基础。

## Resumo

A escassez de água é um problema crítico em todo o mundo, e o Chile não é exceção. Desde 2010, o centro do Chile enfrenta uma crise hídrica contínua devido aos efeitos combinados de uma forte seca e do uso excessivo dos recursos hídricos, especialmente das águas subterrâneas. As comunidades rurais têm sido fortemente impactadas, principalmente porque os poços de abastecimento de água potável apresentam uma queda dramática no nível de água, e alguns até secaram. O cenário de escassez de água requer a integração de atores e disciplinas para aumentar a conscientização sobre as águas subterrâneas. No entanto, como tornar este valioso elemento visível para a sociedade é uma questão que permanece aberta ao debate. Este artigo descreve e reflete sobre o processo de produção de material educativo sobre águas subterrâneas e escassez de água para crianças para promover a conscientização pública. Com base em processos transdisciplinares e coprojetados, este trabalho descreve as percepções sociais das águas subterrâneas entre crianças e líderes comunitários, bem como a forma como a informação científica e o conhecimento local sobre a escassez de água podem ser integrados num livro para a população jovem. Esta pesquisa conclui que projetos educacionais sobre recursos hídricos subterrâneos aumentam a conscientização das pessoas sobre o papel desse recurso oculto no ciclo da água. Esses projetos incentivam a criação de materiais fundamentados e contextualizados que incorporam o conhecimento e a experiência já presentes nas comunidades, aumentando a conscientização pública sobre o papel das águas subterrâneas e os problemas associados à escassez de água, integrando assim a academia e a sociedade. Essa abordagem pode ser uma ferramenta para estabelecer as bases para enfrentar com sucesso a crise hídrica no Chile ao longo de gerações.

## Introduction

Water is an essential element that allows the proper development of human well-being and socio-economic activities. Due to anthropogenic pressures and climate change, surficial water has become scarce in some regions of the world, such as Northern Africa, the Near East, the Western United States, Central and Southern Asia, and the west coast of South America, making groundwater increasingly relevant for water supply (FAO 2021). Even though that context has led to an increasing reliance on groundwater (Megdal 2018), less attention has been placed on this vital, but at the same time, invisible element. Indeed, groundwater plays a relevant role in arid and semiarid regions where surfacial water is scarce or null (Gehrels and Gieske 2003; Siebert et al. 2010; Bierkens and Wada 2019; Gleeson et al. 2020).

Given such conditions, it is increasingly relevant to raise public awareness of groundwater—being an invisible element, groundwater is not well known among the public and policymakers. Indeed, public awareness is a pivotal factor in explaining the success and effectiveness of hydrological practices and projects (Ekmekçi and Günay 1997). In this sense, most recently, the United Nations decided on groundwater as the “2022 World Water Day” theme to make visible this invisible resource found beneath our feet (UNESCO 2022). Nonetheless, little research has provided a reflection and analysis about how to increase public awareness of groundwater, and its relevant role in the water cycle remains an open issue. Raising public awareness of environmental issues has the potential to impact and contribute to developing a good environmental attitude towards environmental protection (Chawla and Cushing 2007) and a sense of concern, responsibility, and motivation to perform responsibly for the environment (Korhonen and Lappalainen 2004).

One way to increase awareness is to augment groundwater literacy among the population, mainly because groundwater is perceived with several misconceptions and knowledge gaps—for example, groundwater is seen as an isolated section disconnected from the surficial components of the hydrological water cycle, such as rivers and lakes (Assaraf and Orion 2005). Dealing with such misconceptions is a pivotal component in the work of hydrogeologists, but even so, misconceptions remain in some legal water management documents. Misunderstandings also exist within water governance authorities, making water resource awareness a matter of extreme urgency among different actors. Further, it is often thought that groundwater is not affected by activities on the land surface, and in addition, aquifers hosting groundwater are sometimes seen as lakes or underground pools instead of water inside small pores (Dickerson and Dawkins 2004). At the same time, policymakers and practitioners manage only basic information about groundwater, impacting negatively on how they evaluate and prioritise projects on the matter (Ekmekçi and Günay 1997).

The lack of public awareness of groundwater and water scarcity is connected to another matter—the challenge of suitable interaction among scientists, policymakers and society to address water problems. Maheshwari et al. (2014) state that suitable integration of disciplines and societal factors promotes the development of trust among the relevant actors. Indeed, a critical barrier to developing effective and sustainable environmental planning strategies is the exchange of knowledge among practitioners and communities (Boreux et al. 2009). Nevertheless, this integration presents several challenges. In some cases, interdisciplinary projects do not deeply integrate areas or approaches, but rather work in parallel isolation with researchers working from within their own disciplines (Martin 2020). Rangecroft et al. 2021 suggest that geoscientists need to integrate social considerations with specific training in power asymmetries, ethics, cultural differences, and communication barriers before participating in interdisciplinary initiatives.

To tackle the lack of public awareness of groundwater, one must take into account the two arguments mentioned before—lack of groundwater literacy, and lack of spaces where researchers from different disciplines and nonacademic actors work together. This study conducted an educational project in Central Chile, a territory where water scarcity and resource issues are growing steadily. Water conflicts and severe drought have marked this semiarid climate region and there is an increased demand for collaborative initiatives.

In this context, a project was initiated with the aim of making a children’s book using transdisciplinary and co-design tools as participatory methods, in an effort to build bridges between different disciplines, including academia and grassroots actors. The project described in this paper aims to address information gaps and promote a better understanding and public awareness of groundwater and water scarcity. This paper has two goals—the first one is to describe the social perception of children and community leaders with respect to groundwater and water scarcity, while the second one is to analyse how making a book based on the integration among actors, using transdisciplinary and co-design tools, could promote a better understanding of groundwater and water scarcity themes.

This project took into account the promotion of groundwater literacy, under the premise that the praxis of making a book as a science communication tool is an instrument for democratising scientific knowledge, promoting awareness, opinion and understanding of science, as well as producing dialogue among the different people involved (Burns et al. 2003). Additionally, the process of bookmaking itself addresses the status of current and future knowledge (Gilbert et al. 1999).

Further, the project took stock of the potential for the integration of actors and perspectives around water scarcity and groundwater. From its inception came the concept of a space where grassroot organisations and scientists from different disciplines can come together to use transdisciplinary and co-design opportunities for filling the gaps between the scientific and societal spheres. Transdisciplinary (Td) is understood as the integration of academics and actors who are not part of academia, allowing the integration of ideas from different cultural perspectives (Bergmann et al. 2021; Krueger et al. 2016). This approach looks into “*stressing diverse disciplinary perspectives that have to be reorganised for societal problem solving*” (Pohl et al. 2021). These kinds of projects include participatory design and tools to integrate approaches and perspectives.

The book’s development was focused on children rather than their caretakers or teachers because children have had limited participation and opportunities to integrate environmental projects but are profoundly affected (Hill and Butterfield 2006). Also, according to the Sendai Framework for Disaster Risk Reduction (UNDRR 2015), children have a vital role in strengthening community resilience and in being agents of change to promote a more sustainable future. In an increasingly developed world, some scholars suggest that children, especially those living in big urban cities, do not have meaningful opportunities to interact with nature and thus develop an emotional distance from it (Orr 2004). Thus, in the context of climate change, there is an urgency to include children in participatory projects about environmental issues (Rousell and Cutter-Mackenzie-Knowles, 2020). In this context, Wells and Zeece (2007) argue that children’s books can effectively promote children’s understanding of their environmental context, and this project seeks to advance that line of reasoning.

This paper is organised into four main sections, whereby the first part introduces the context in which this initiative took place, emphasising why an educational and transdisciplinary project is particularly relevant in Chile. The method is described, and then the paper depicts the project’s results, illustrating how the book was designed and changed by the integration of actors.

## Methodology

### Situating the context: a brief review of the Central Chilean water scarcity issue

Water conflicts have increased in number and size in Chile, as in many parts of the world. Both academic and state reviews have acknowledged this situation in Chile (CNID 2016; Delamaza et al. 2017), identifying the fact that water stands as one of the main reasons among several sources of conflict. These conflicts arise around two overarching sets of problems. In the north of the country, there is overexploitation by mining activities of fossil and nonrenewable groundwater resources (late Pleistocene and mid-Holocene mean ages; e.g., Viguier et al. 2019). In the center and south of the country, conflict is related to several activities that have triggered surficial water and groundwater depletion—for instance, the interventions on rivers for hydroelectric generation, farming practices to produce avocados, and agricultural pollution from the fertiliser overuse (e.g., Bauer 2005; Fernández et al. 2017; Taucare et al. 2020; Barría et al. 2021; Fuentes et al. 2021; Madariaga et al. 2021). The conflicts have arisen due to the allocation of water being conducted without a rigorous scientific and technical assessment, prioritizing the water supply instead of regulating the water demand. During the 1980s, the Chilean military dictatorship promulgated a new Constitution and Water Code establishing a water market where water is treated, in practice, as a completely private good under laissez-faire principles (Bauer 2005; Harris and Roa-García 2013; Prieto et al. 2019). However, it has been shown how the legislation’s focus on surface water and the omission of a conflict-resolution mechanism has deepened the problems (Bauer 2005, 2015). After the first reform (in 2005), a new reform measure aiming to address these issues was approved in 2022, but it has yet to be fully implemented.

As aforementioned, the Water Code regulates water extraction in Chile (Congreso Nacional de Chile 1981). It is a free-market water-management model based on a “water rights” system, whereby water is considered private property and tradeable. A “water right” corresponds to the permission to extract a given quantity (in volume per unit of time) of either surficial water or groundwater from a determined point for its exclusive use. In other words, a water right is equivalent to authorised water extraction. The water right is perpetual upon registration in the Real Estate Registry as private property. Although, when requesting a groundwater right in restricted areas (i.e., groundwater-scarce areas), it is provisional and not registrable in the Real Estate Registry, being revocable during high-water stress periods. A water right can be classified as conjunctive or nonconjunctive, depending on whether the water is totally consumed or returned to its source after its use, respectively. How the allocated discharge is used depends on the exercise of the right, which results from a combination of the probability and the timeliness of the use of the right. According to the probability of use, a right is permanent or eventual. The first one grants the holder the total allocated discharge use, while the second allows the allocated discharge use solely in case of surplus water from a given source after the permanent rights have been supplied. According to the timeliness of use, a right is continuous, discontinuous, or alternated. The continuous right grants uninterrupted water use during the whole day. The discontinuous right grants water use within specified and pre-established periods. The alternate right grants water use by turns between two or more holders. Considering the groundwater use in the Central Chile macro-region, more than 99% of water rights are conjunctive and permanent-continuous (according to the Central Chile basins’ water rights recorded in the Dirección General de Agua database). That means that 99% of groundwater extractions are authorised for total and uninterrupted use during the whole day, every day of the year. In addition, more than 90% of the allocated groundwater rights are perpetual (according to the Central Chile basins’ water rights recorded in the Dirección General de Agua database). Counterintuitively with the water regulation basis, perpetual rights have also increased over time in restricted areas.

In terms of contextual aspects, Central Chile (~80,000 km^2^) has a semiarid climate and hosts ~12 million inhabitants (~70% of the Chilean population). This region has been experiencing an uninterrupted sequence of dry years since 2010 (precipitation and river discharge deficits up to 45 and 90% respectively, according to Garreaud et al. 2017, 2020). Given the notable decreases in surficial water, the authorised groundwater extraction has significantly increased to supply socio-economic needs (e.g., Taucare et al. 2020; Figueroa et al. 2021), primarily to support large-scale farming activities (Anríquez and Melo 2018; Novoa et al. 2019; Webb et al. 2021) such as avocados, which require >210 m^3^/day/ha (Madariaga et al. 2021). The major groundwater supply source in this zone corresponds to the Central Valley alluvial aquifers (according to the Dirección General de Agua database). Regardless of the importance, the authorised groundwater extraction (under the concept of water rights) has progressively increased from 21.11 m^3^/s in 1980 to 222.86 m^3^/s in 2010 and even reached 405.50 m^3^/s in 2022 (according to the Central Chile basins’ water rights recorded in the Dirección General de Agua database). Consequently, an alarming decline in groundwater levels of the alluvial aquifers reached ~40 m in the 2010–2020 period (e.g., Figueroa et al. 2021). Water infiltration from agricultural areas using fertiliser and pesticides has impacted groundwater quality by increasing the concentration of NO_3_, Cl, and other trace elements (Fernández et al. 2017; Arumi et al. 2020; Taucare et al. 2020). It is important to note that before the 2022 reform, groundwater extraction approval was basically subject to administrative requirements rather than hydrological or ecological aspects.

Despite water scarcity being an acute problem, policy and research projects have targeted water issues, mostly adopting what has been called a “top-down” approach and little research has provided integrations, especially from the point of view of the water-stress-affected users, organisations and/or communities living in specific territories (Urquiza and Billi 2020). At the same time, the importance of groundwater is barely touched in the school curricula, as is the case in other semiarid regions (Hussein 2018). In the Chilean school curricula, groundwater is slightly included in the contents and learning objectives of the areas of natural sciences and history, geography and social sciences (levels from 9 to 12 years old). The curricula bases and the school study programs open up the possibility of working on a groundwater and water scarcity theme, which is consistent with the learning objectives, but groundwater and water scarcity are not deeply developed in the current curricula or textbooks. In this sense, this project tackles the gap in groundwater materials and in children’s education.

### Participants

The project was conducted in 2021 by an interdisciplinary group of six researchers, including social scientists, a hydrogeologist, an educator, an artist, and a communicator. The participants from the societal group included 40 pupils from public schools (a rural one from Putaendo, and an urban one from Santiago), one community leader from a nongovernmental organisation, one from the National Federation of Rural Drinking Water Associations (FENAPRU) and seven school teachers. The participants contributed within different frameworks in the three phases of the process.

The procedure of making the book was organised in three linear phases: (1) predesign, (2) testing the book prototype, and (3) measuring the impact. The goal of the first phase was to explore the perception of groundwater and water scarcity issues by societal actors to build the thematic book framework. In this stage, 25 children from 8 to 12 years old and two community leaders were involved. This stage sought to answer the following exploratory research questions: (1) How do societal actors—children and community leaders—perceive groundwater resources and water scarcity? (2) What kind of information gaps do societal actors have? (3) What kind of actions do they promote to face water scarcity?

The second-stage goal was to build a book prototype and present it to societal participants, and to receive feedback from them. This phase took place 3 months after the first phase, and the participants were the same as in the previous stage. The book was revised according to the collected feedback.

Finally, the third phase aimed to explore how the final version of the book promotes public awareness and delivers more comprehensive knowledge of water scarcity elements, testing the material with a broader audience. In this stage, a group of 15 pupils (from 8 to 12 years old) participated, who belong to the same schools, but were not part of the prior phases, plus a group of seven teachers from different schools.

### Procedures

The tools chosen to gather information and integrate societal actors included several qualitative methods. Overall, in the three stages, online workshops were conducted (a max of eight participants in each meeting) from January to October 2021. Although they were planned as traditional workshops, they were adapted into an online format due to the COVID-19 pandemic of 2020–2022. In Chile, the classes of most schools remained online in 2021.

In the first stage, where the goal was to explore perceptions of societal actors in order to build the thematic framework of the book, in the case of children, drawings as a qualitative method were used to explore both social and risk perceptions of groundwater and water scarcity. Drawing has been commonly used to get a psychological perspective to explore environmental conditions around children (Blades and Spencer 1994), and it is a great strategy for participants that may have difficulty expressing their thoughts verbally (Ongena and Dijkstra 2007). Those drawings (25 in all) were evaluated through inductive analysis in order to understand drawing contents and explanations (Table 1). Because this project was conducted in the middle of the COVID-19 pandemic, the research team sent a box with school supplies for drawing, comprised of pens, paper, and plasticine, to the participants’ homes. Their teachers and parents gave them the following instruction:

- Activity name: Draw what comes to mind. We invite you to make two drawings on white paper with the materials included in the bag delivered to your home/school or with the school supplies you have at home, answering the following questions: (1) What does the hydrogeological water cycle look like? (2) What do you think the water looks like and how does it move under your feet (underground)?

**Table 1:** Description of the three phases of the project.The tools used  are included in the ESM electronic supplementary material

Parameter	Phase		
	Predesign	Co-designing	Measuring the book’s impact
Goal(s)	To explore the perception of groundwater and water scarcity issues by societal actors, focusing on pupils, to build the thematic framework of the book	To receive feedback from participants	1. To explore the perception of the final version of the book with a broader audience. 2. To assess comprehension of the book content
Data collection methods and data analysis	Eight online workshops with pupils (drawings and open-ended questions). One multistakeholder discussion group with a natural science researcher and a community leader facilitated by the social science researchers (story wall). One in-depth interview with community leaders (open-ended questions). The information provided by the drawings was analysed by a thematic checklist (see details in the ESM) and observation records. Notes were taken and the activities were recorded. For the discussion group and interview, notes were taken and the activities were recorded. Inductive and thematic analysis were included in both cases to reply to the research questions	1. Six online workshops with pupils 2. In-depth interviews with community leaders. Open-ended questions were conducted to identify their opinions about the book. Notes were taken and the activities were recorded. Inductive and thematic analyses were included	Online questionnaires (see more details in the ESM)
Participants involved	Total of 25 pupils (8–12 years old) and 2 community leaders	Total of 25 pupils (8–12 years old) and 2 community leaders	Total of 15 pupils (8–12 years old). A new group from the same school and 7 school teachers
Timing	4 months	6 months	1 month

Two instruments—observation record and checklist—were used to evaluate the children’s drawings and their comments about them. In the first case, the observation record allowed for building a narrative of the pupils’ explanations about their drawings. Secondly, the goal of the checklist was to measure the presence or absence of the evaluation indicators, which were the stages of the hydrogeological water cycle, the identification of water uses and the explanation of water scarcity—see more details in Table S1 in the electronic supplementary material (ESM).

Additionally, two methods were used to understand social concerns and perceptions of community leaders. First, a multistakeholder discussion group tool was implemented to integrate participants’ perspectives, while discussing their feelings and main concerns on water scarcity issues (Fry 2021). This activity was conducted by a natural science researcher and a community leader and facilitated by social science researchers. This method enables actors to meet and exchange their experiences in a balanced way, promoting effective co-production of knowledge (Fry 2021). In this activity, a story wall tool from the Swiss Academies of Arts and Sciences’ td-net toolbox was used (Wülser 2020). This method, which collects individual perspectives, promoting the understanding of the current water scenario and past events (Wülser 2020), allows participants to draw a memory timeline. In this case, local leaders drew on white paper and highlighted some events of the Chilean water-management historical lines. At the same time, to complement this information, an in-depth interview was conducted with another community leader, who was not able to attend the stakeholder discussion group. All the gathered information was thematically analyzed in order to answer the research questions.

The second phase—testing the prototype—included six co-designing online workshops with the same participants (pupils and community leaders) as the predesign stage. The book prototype was presented (images and text) via given activities, and an open discussion was conducted to comment on the material. Later the feedback was reviewed, and the book was revised accordingly to their opinions. Participatory observation was conducted in order to register the changes and opinions, and the workshop proceedings and outcomes were recorded. Subsequently, the notes were thematically analysed in order to address changes in the prototype.

Finally, the third phase of the process—measuring the impact—took place 3 months after the co-designing process. In pursuit of the goal of testing the material with a broader audience, 15 pupils, who did not participate in the prior process, read the book and then responded to an online questionnaire (see more details in Table S2 in the ESM). The questionnaire included five sections. Open-ended questions, true or false, and alphabet soup activities were included. These questions aimed to assess comprehension of the book content, specifically the understanding of the key concepts of each chapter of the book.

At the same time, to expand the perception of the book, seven school teachers were invited to reply to an online questionnaire after reading an online version of the book. Five open-ended questions about the design, content, and elements of the book useful for their teaching activity as well as suggestions for improvement and strengths were included. Finally, space for free comments on the book was added (see more details in Table S3 in the ESM). Table 1 shows the entire research process.

Note that, as per the project objectives, a predominantly qualitative approach was pursued, as it is better able to interpret the participants’ intended meaning and fit the emergent design inherent in a book-making process. This approach focuses on particularity rather than generalisations, aligning with the aim of creating an impact with respect to two particular goals.

## Results

This section includes the main results of each stage of the process. The first part describes the perceptions of the social actors regarding water scarcity and groundwater and how the book’s conceptual framework grew by integrating their views. The second part of this section presents the book and how it changed during the co-designing process; additionally, an effort is made to describe the co-designing process and how the information gaps were filled. The last part highlights how the book was perceived, and how it promotes public awareness and comprehensible knowledge of water scarcity elements among a broader audience.

### Social perception of groundwater and water scarcity

#### Young population

**Fig. 1:**
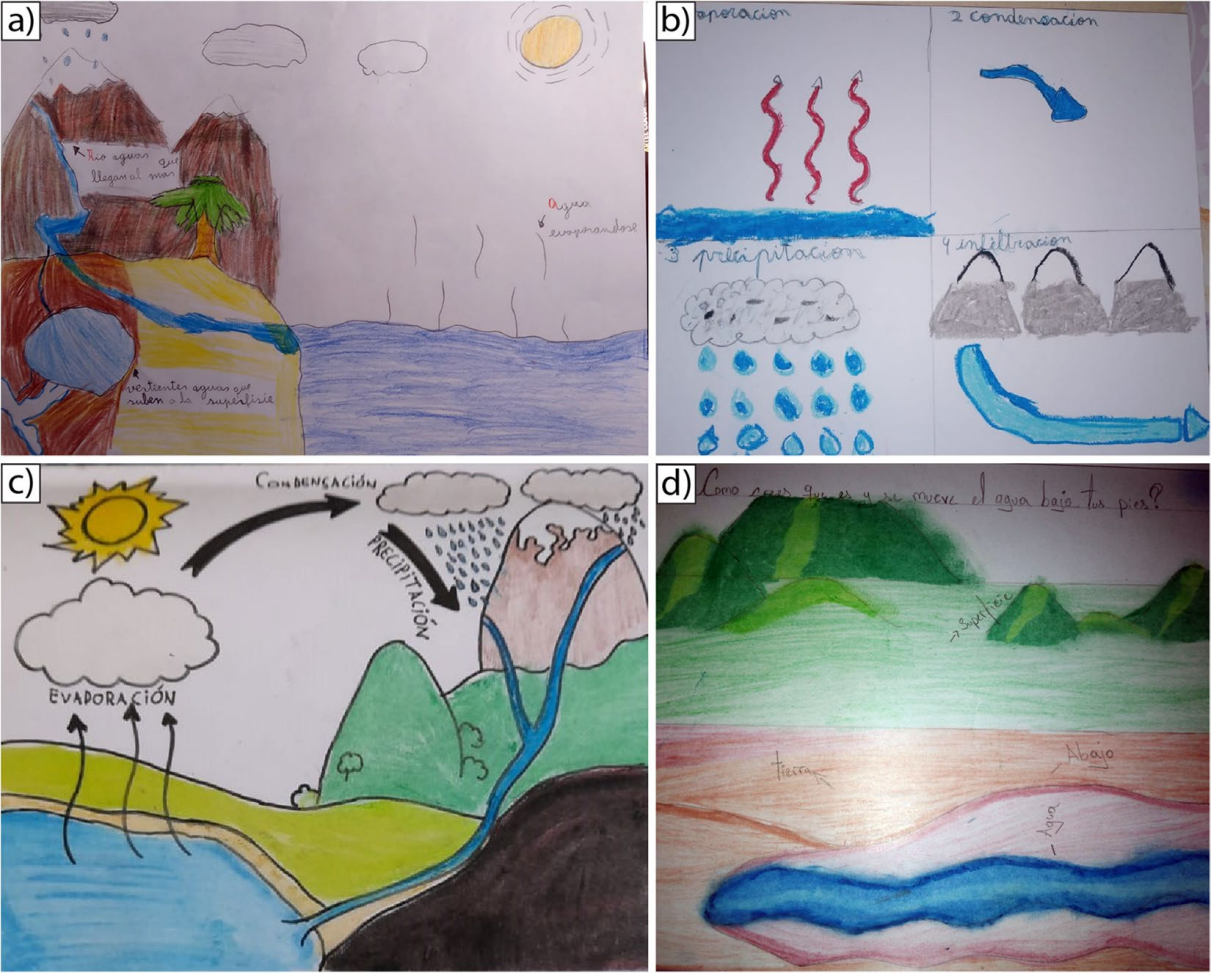
Four examples of participants’ drawings of the hydrogeological water cycle (**a**–**c**) and groundwater (**d**)

In terms of the hydrological water cycle, overall the pupils managed to provide basic information about the stages, but they identified the water cycle process with beginning and ending points. In other words, they did not see the water cycle as a cyclical process. We could observe this issue in their oral explanations: “First the water is in the ocean, then it evaporates and becomes clouds, then it falls as rain or snow. Then part of it stays in the mountains as snow, and the process ends when the other part goes down the rivers and reaches the sea” (pupil from an urban school). At the same time, in their drawings, an absence of subsurface interaction was observed (see Fig. 1). Runoff and infiltration were not included in the hydrological water cycle (84% of their drawings). Another absence was groundwater. In general, they did not include groundwater in their representations (96% of the drawings), and when drawn, it looks like rivers and lakes.

In terms of water scarcity, qualitatively, it was observed that some pupils were aware of the situation depending on where they lived. Participants from Santiago—an urban city and the capital of Chile—were not well aware of the issue. However, participants from Putaendo, a rural city, were well aware of it. This point could be observed in the quotes below:

*We recycle water every day with my family, we reuse the water from the washer to water plants because the water is scarce*. (12-year-old participant from a rural school)

*Putaendo is dry*, *there is a river in front of my house that does not bring any water*, (11-year-old pupil from a rural area)

*It does not rain much anymore*. *I notice the lack of water because in my house sometimes there is no water and sometimes there is… In the park, before there was grass and now everything is dry…*. (12-year-old pupil from a rural area)

*We don´t have any problem with water. We have water at home and in the school*. (12-year-old pupil from an urban area)

We observed that some pupils also demonstrated an empirical understanding of water justice, in the sense that some of them explained that point. The following quote illustrates this idea.

*There is still a lot of water on the top of the hills. The problem is that some people redirect the river on the top and they take the water for themselves, so in the town, we don’t get it. This is not right because the water is just for some people, not for all the town.* (10-year-old pupil from a rural area)

The preceding point was mentioned by several pupils who also included “the monoculture” concept in their explanations. This level of understanding was not observed in participants from Santiago, although, in both places, pupils were able to reflect on the importance of the water.

In line with the results about the hydrological water cycle, an important point on water perception is that when they mentioned something about water scarcity, they meant only surficial water. Groundwater was not part of the discourse. They described water scarcity by the element they could see in their town, for instance, the dry rivers. Mostly, groundwater, infiltration and aquifers are not concepts included in their descriptions, and when those concepts are integrated, they look like underground rivers and underground lakes (shown in Fig. 1d).

In terms of actions, young participants pointed out several activities to address the water issue. However, they were all at an individual level without mentioning either community or officials’ responsibilities regarding this matter. In this sense, daily actions such as turning off the water while brushing their teeth or taking short showers were mentioned. While pupils from rural schools mentioned family actions like reusing washing machine water or saving rainwater, the participants from urban schools were less aware of said activities.

Another finding was that, in general, pupils used water scarcity and drought as synonyms. They were not able to describe orally any difference between those two concepts, as is well described by this quote:

“*We have a big problem with the drought because the rivers are dry due to the people who redirect the river, so people in the village don’t have water at all*” (12-year-old rural school pupil).

#### Community leaders

The perception of community leaders about water scarcity issues was highly connected to the political aspects of water management in Chile. Since the economic and political context have dramatically changed over the years, the impacts of those changes on the groundwater were highlighted by these actors. They emphasised the importance of prioritising water as a human right over private interests.

One of their first insights was the idea of changing the way of perceiving and understanding water, as well as speaking about it. This point is illustrated in the following quote:

*Water should be understood as an element and not as a resource, because when we see it as a resource, it supposes that water is seen only through an economic lens. I always suggest using the word ‘element’ rather than ‘resource’. We have to change the way we relate to the water and how we speak about it.* (participant from the FENAPRU)

Although the community leaders acknowledged that, in some rural territories, there is a low level of information about groundwater, they suggest the situation is worse in urban cities. According to them, people from rural territories see how and when the drinking water wells experience water level drops, or even dry up but, in urban cities, the situation is not experienced. This point is reflected in the following quote:

*Commonly, people turn on the tap, and water comes out, but they do not ask about how water got there. If there is water there is not a problem. The problem is when the wells are running dry and that situation currently takes place only in some places*. (participant from a nongovernmental organisation, NGO)

At the same time, they highlighted water issues as an opportunity for local governance. “*One of the key aspects is to have the opportunity to manage the local element and local needs by local people*”, a participant argued. In this sense, they addressed the need to integrate different kinds of water governance into the national system.

The prevalent feeling when they talked about water was that of injustice. They approach water governance from a critical perspective, claiming urgency to change the role of the state over the private sector. In order to improve awareness, they also highlighted the importance of including rural elements of water scarcity such as water trucks and water storage systems in the book. Also, they suggested the need to explain how water scarcity is visible in urban cities, for example in dry parks. The following quote illustrates this point: “*In Santiago you can see how much richer people are or the municipality is by how greener their parks are. The lack of access to water is visible, but you have to look carefully*” (participant from an NGO).

In terms of the hydrological water cycle, participants pointed out that, though this information is managed in general, it is not well known how human activities interact within that cycle. At the same time, they mentioned another gap: “*Some people think that the water is lost once it reaches the sea. There is no clarity about the ecosystem perspective and what the role of groundwater is in this context*” (participant’s quote from an NGO).

#### Summary of different water scarcity perceptions

**Fig. 2:**
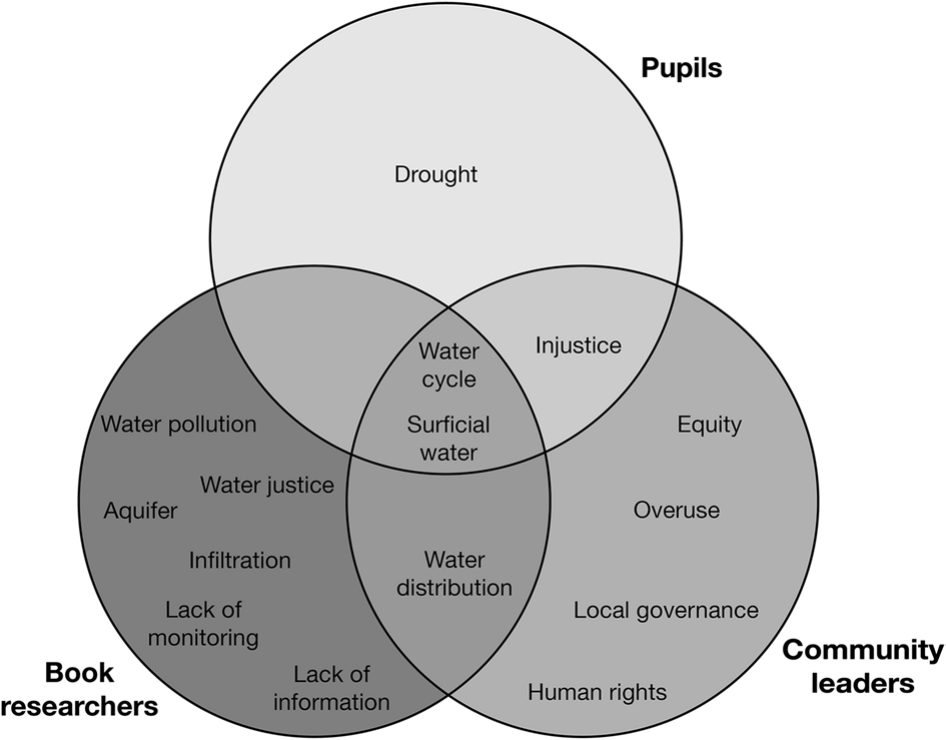
The initial ideas of the researchers developing the book with the complementary information collected from pupils and community leaders. Most relevant concepts were highlighted by each actor

Figure 2 contrasts the initial ideas of the researchers developing the book with the complementary information collected from the pupils (see section “Young population”) and community leaders (see section “Community leaders”). One of the main goals of the book project was to increase public awareness of groundwater, but throughout the process of integrating the societal actors, the thematic framework of the book was extended to include the political and social aspects of water governance, even though it was originally inclined towards the natural sciences. Figure 2 shows the most relevant concepts highlighted by each actor. The interaction of the societal actors’ concepts, such as human rights and equity, both highlighted by the community leaders, were included in the thematic framework of the book.

### The book and how the prototype was changed

This section describes the final book version and how it was collectively transformed by the integrative process and how the information gaps were filled. Overall, as the prior section described, the thematic framework was extended by including concepts such as water governance, human rights and equality. The book’s pictures were also changed according to the collected feedback.

The structure of the book has four chapters: (1) hydrological water cycle, (2) groundwater, (3) water scarcity, and (4) individual and community actions. As a complementary section, the book includes a games section to activate the learning process. The Table 2 describes the main concepts integrated in each chapter and the knowledge gaps addressed. The book also integrates a comic strip as an educational and didactic tool allowing for a fluent and fast interpretation, making it more accessible to any type of reader. The comic includes a story about a group of characters who one day wake up and find there is no water in the town, thus they look for answers and they start a journey to understand what is going on. The story was divided and presented in each chapter of the book. The final version of the book “*Agua: una travesía infinita*” (translated as “*Water: an (in)finite journey”*) is downloadable (see Vargas-Payera and Ramirez 2021).

**Table 2:** The main concepts integrated in each chapter and the knowledge gaps addressed

Book aspect	Book chapter			
	Chapter 1: Hydrological water cycle	Chapter 2: Groundwater	Chapter 3: Water scarcity	Chapter 4: Actions
Chapter contents	Focus on expanding the definition of the water cycle, and making efforts to explain infiltration and how social activities impact this cycle	Focus on what groundwater looks like, how it moves and accumulates, age, and how people use it, and pollution issues	Focus on explaining what is water scarcity vs. drought, paying attention to how social decisions impact water governance	Focus on actions to mitigate water scarcity; special efforts were made to promote collective practices. Description of three examples of people who work on protecting water (academia, social rural governance and NGO representatives)
Gaps addressed	- Infiltration as a part of the hydrological cycle. - Hydrological cycle as a continuous process, without ending	- Groundwater and aquifer concepts	- Differences between water scarcity and drought. - Gender issues	- Community actions

#### Book changes

In terms of the integration of societal perspectives, the main changes were made by bringing in (or integrating) the pupils’ points of view. In this section, how the change process took place is described by the contrast of the prototype and final images.

##### Cover and water governance

**Fig. 3:**
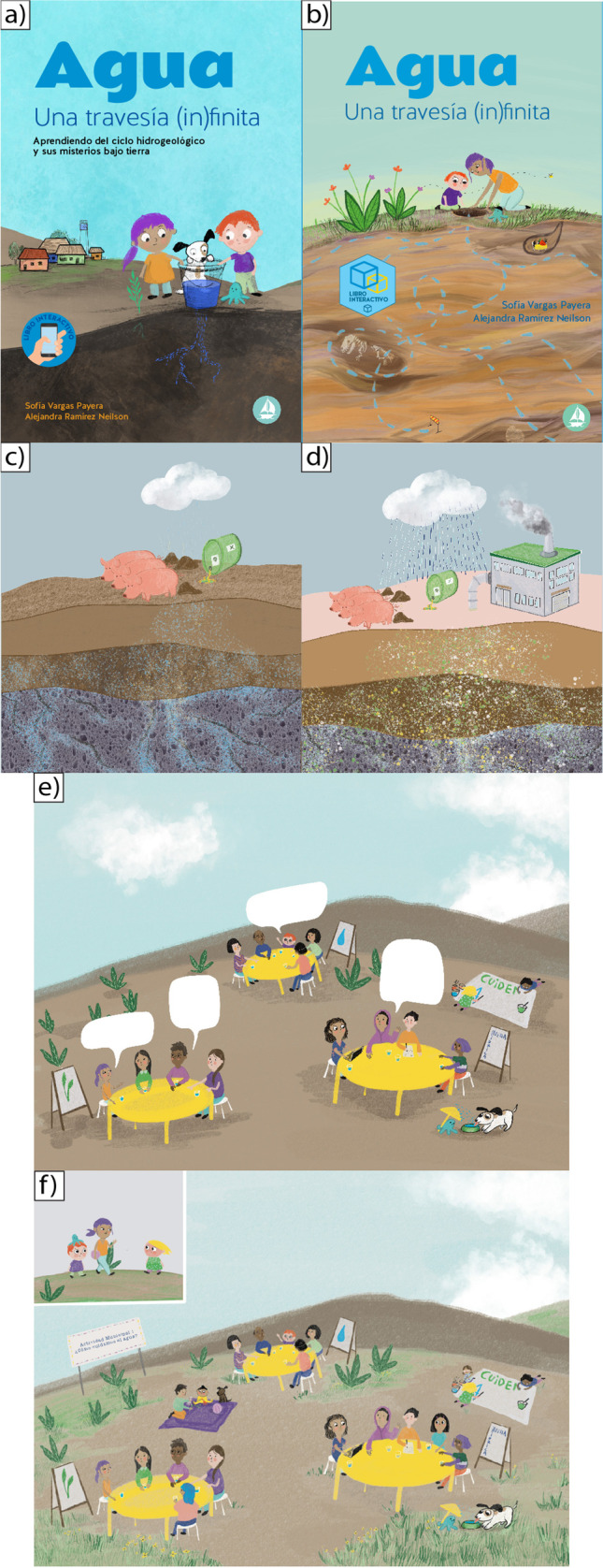
Illustrations of the book changed by participants’ observations. Images showing the prototype (**a**,** c**,** e**); images showing the final versions (**b**,** d**,** f**) to see the final version of the book, please visit www.travesiafinita.cl

The goal of the cover (Fig. 3a,b) is to captivate the attention of potential readers. In this sense, the cover was designed together with a young population not only to describe the issue but also to get the reader’s attention. At the suggestion of the pupils, the final version (see Fig. 3b) includes characters that look older. For the participants aged from 8 to 12 years old, in the first prototype the characters looked “too young” (see Fig. 3a); thus, they did not feel represented. In this sense, the main character was changed to a 10–12-year-old girl, while the other character remained at 5 years old. Overall, participants suggested that the cover must look positive. Taking into account that suggestion, the idea of showing the underground was retained, but the impression was changed by using colourful tones and including vegetation to provoke a more positive atmosphere. Water governance (Fig. 3e,f) is one of the key concepts developed throughout the book. In this sense, community and local organisations are described as ways to address water scarcity. In the prototype (see Fig. 3e), an illustration where all the characters are discussing ways to address that topic was presented. Participants suggested that, as well as on the cover, the colours selected and the lack of nature signals a permanent lack of water rather than an option for solutions. They asked for grass and use of colourful tones (see Fig. 3f).

##### Water pollution 

The young participants asked for inclusion of a factory in the illustration about water pollution (Fig. 3c,d). This request was because “*it is important to see who pollutes the water*”, as said by a participant from the rural school, which was understood as a call for attention to the importance of concrete language in science communications. At the same time, some young participants suggested showing more of the impact of contamination on aquifers, which is why stronger colours were used in the final version (see Fig. 3d).

Another change suggested by young participants was the integration of a grandmother as a character in the book. This figure was described by pupils from the rural school as a person who addresses the water issue in their families, a person who has a great amount of local knowledge. In the comic, a grandmother was the person who introduced the conceptual difference between drought and water scarcity and how the landscape has changed in the last decades. They also promoted the integration of animals, as beings who are affected by the lack of water, but who are not always considered. This point is reflected in this quote: “*In the last years we’ve seen how animals have died from lack of water, all animals need water*” (pupil from a rural school). This point was taken by including a dog and pigs in the comic story. The end of the story, where a dog is illustrated as drinking water, suggested by the pupils, shows that by protecting water there are good outcomes.

At the same time, community leaders promoted the integration of local rural elements such as cistern trucks and rural drinking water systems in the book, both of which are not common in Santiago, the urban capital of Chile. Those elements were included in order to show the different realities. At the same time, the pupils from rural areas pointed out the idea of including several ways of reusing water, such as using the water from the washing machine for watering plants. Those ideas were included in the last chapter.

In terms of addressing gaps, the book includes the concept of the hydrogeological water cycle. The decision to include the geological elements of the process was designed to emphasise infiltration processes and groundwater. Due to the lack of appropriate understanding of concepts on the relationship between rivers and groundwater, special effort was made in the illustrations and text to explain that water moves through sediments and rocks. Chapter 2 has three illustrations to depict that point. The narrative includes the water in the social-ecological system, describing how surficial water starts to move underground, becoming groundwater, and how it interacts with human and societal activities. Several analogies were used to describe how water moves underground, for instance, to describe a fracture, the image of a wall fracture was included, and a sponge to wash dishes was used to describe an aquifer. In terms of water governance, special effort was made to explain how water is used and who uses it.

### Post assessment: perceptions of the young population and school teachers about the final version of the book

This section describes the main results of the questionnaires applied to pupils who were not part of the co-designing process, but were students at the same schools (the questionnaire is included in the ESM). The goal was to understand the comprehension of the book content and general perception of the book as a tool to increase public awareness. After replying to an online questionnaire (more details in section “Methodology”), overall, the young population gained knowledge about groundwater, highlighted by the level of correct answers in the chapters about water scarcity and actions (see Table 3).

**Table 3:** Number of questions answered correctly in terms of book content comprehension. The online questionnaire was applied to 15 pupils who were not in the co-design process (see ESM)

Criteria	Percentage of correct answers
Per school level	
6th grade (11 years old) (*n* = 7) 5th grade (10 years old) (*n* = 10) 4th grade (11 years old) (*n* = 8)	74% 69% 69%
Per book chapter	
Chapter 1: Hydrological water cycle Chapter 2: Groundwater Chapter 3: Water scarcity Chapter 4: Actions	79% 77% 84% 94%

In terms of their perception of the book, in the open-ended questions section, pupils highlighted that the book was attractive due to the great number of illustrations and drawings and the comics. At the same time, they liked the second chapter the most because “*we could see how the water moves, and how the aquifers are. Although I would love to see exactly where they are*”, a participant from the urban school said. Among the information described in the book, some participants highlighted that the gender information, such as that women are affected more than men in the water scarcity context, and information about the water footprint, e.g., how much water is needed to make a t-shirt, were the most striking details described in the book.

In terms of how the book impacted the level of pupil comprehension, it is interesting to note that greater achievement (in terms of correct answers) was expected for the first chapter about the hydrogeological cycle, since the water cycle is part of the school curricula, thus pupils have become quite familiar with those themes. However, the number of questions answered correctly in terms of book content comprehension was higher for the third and the fourth chapters (water scarcity and actions).

In terms of the school teachers’ perception, their answers indicated that the book could be useful for pedagogic purposes (see questionnaire in the ESM). According to them, the main strengths of the book are: (1) the diversity of child characters in terms of ages and physical features; (2) the promotion of critical thinking about water scarcity issues, (3) providing scientific information on the groundwater and societal concerns with attractive images and direct explanations and (4) the integration of different subjects, such as science, citizen participation, and environmental themes. The school teachers commented that there is room for improvement in terms of (1) expanding the game section; (2) including all the comic strip sections together—which were added at the end of each chapter—in order to promote a better understanding of the cartoon story, comprehension and cohesion, and (3) to include more questions, especially more exploratory questions.

The following quotes illustrate those points:

“*I like the book because each chapter works well separately and all together. The content is attractive but deep enough. I also highlight the order of the chapters: beginning with science information, then awareness of social issues and ending with encouragement for actions and citizen participation. I find the learning assessment section valuable and useful”. *(female art teacher from Santiago)

“*The improvements could be in the line of including more activities and games. At the same time, every chapter could add more critical questions, not necessarily including the answer but promoting critical thinking”.* (male science teacher from Santiago)

## Discussion

This paper highlights the process of creating grounded and contextualised materials, in the form of a book, that incorporate the knowledge and experience already present in the communities and how it is connected to scientific information in order to raise public awareness of water scarcity and groundwater.

The paper also describes several disconnections. On one hand, there is a distance between children who live in an urban city and water scarcity issues. The lack of water is not yet a concern among the urban young population. This social perception differs for children living in rural areas, who were very active and critical about water scarcity and the need to protect water. As Louv (2008) argues, the proximity to environmental issues affects the identification, level of involvement and perception of the problem. For instance, children around 5 years old in Central Chile have hardly seen rain in their lives, compared to children over 12 years old.

Another disconnection highlighted by this process was between the young population and groundwater, being an element not visible, not included in their narratives nor included in their school books. In this sense, this project confirms the lack of literacy and lack of appropriate understanding regarding groundwater stressed by literature. The results are similar to findings suggested by Dickerson and Dawkins (2004) about young populations’ understanding of groundwater as lakes or rivers. As Assaraf and Orion (2005) highlight, groundwater is seen as a disconnected element, and the underground is seen as a homogeneous space.

In terms of how the process was developed, keeping the interests of the participants engaged throughout a participatory project is one of the challenges of this kind of initiative. This situation was addressed by making a book, such that the physical end product was a great opportunity, not just to integrate experiences, but also to retain interest until the final stages of the process. When the project was finished and the book was released, and it was possible to observe qualitatively the high level of the participants’ engagement; pupils expressed feeling that they had participated in making “their own book”. One participant (11 years old) in the final event said: *“Because we participated all those months, we feel that we did something to change the water problem in Putaendo. We see our participation in the book*”. This level of involvement was critical to achieving the goal of the project. As Rousel and Cutter-Mackenzie-Knowles (2020) argue, participatory approaches enable children to engage with environmental and climate change issues. The participants of this project were enthusiastic about taking part, which can be understood as a strong need for spaces that bring adults, researchers, and children together outside of the school setting to discuss environmental issues such as water scarcity. This project reveals the value of transdisciplinary tools to engage children with complex and abstract geological concepts such as aquifers, as well as to involve them in critical areas like water scarcity. To confront perspectives allowed one not only to expand the framework of the book but also to promote empathy among participants. A participant (10 years old) from the urban school mentioned “*I didn’t know that it is really dry nearby Santiago and there are schools without water”.*

Another point highlighted by this project was how, through the integration process between community leaders and hydrogeologists, it allowed the incorporation and even the change of some concepts, like “resource” used as a synonym for groundwater. Bracken and Oughton (2006) point out that the utilisation of some concepts by scientists without a cultural background could be problematic, making evident some cultural differences. In this sense, the exercise of designing the book collectively allowed the integration of those narratives and angles. The results of this paper invite hydrogeologists to join these types of projects. As several authors claim, these kinds of interactions allow scientists to present complex scientific concepts to an audience impacted by their studies, engaging societal issues. Those kinds of interaction extend the dialogue between scientists and society, resulting in longer-term and two-ways benefits for practitioners as well as society members (Maheshwari et al. 2014; Rangecroft et al. 2021)

During the process of making a book, children feel greater empathy with content that reflects their reality, both material and socio-emotional. As a female pupil from the rural area pointed out: “*The book represents us. I like that it shows how our grandmother teaches us a lot about how it was in the past when it rained more and the town was different. I love the drawings and understand that women suffer more from the impact of water scarcity than men”.* The children felt more connected with characters from their age group, with a grandmother figure, and with elements that are part of their daily environment. In this respect, Burns et al. 2003 indicate that science communication projects must emphasise applications and implications of science within the local context to promote interest and understanding.

Although the book has a great number of local elements, it includes hydrogeological information about groundwater and the hydrogeological water cycle, and it also introduces elements to understand that a water crisis goes beyond drought. Thus, the book could be used and be relevant to other regions, not only Central Chile.

Finally, a book built as a material product resulting from an integrative process could be considered as a “boundary object”. This concept, introduced by Susan Leigh Star and James Griesemer (Star and Griesemer 1989), claims that this kind of initiative crosses boundaries and facilitates communication and understanding among participants. Subsequently, this book was an interesting creation resulting from a conversation and crossing boundaries among scientists, school communities and community leaders, as well as between children and the adult world.

## Conclusion

Despite the fact that Central Chile has been severely affected by drought, groundwater extraction has increased in the last decade, a setting that urges one to look into raising public awareness of groundwater issues. This paper describes how making a children’s book could be an inspiring process, and also a great opportunity to integrate knowledge and experiences. Especially in the context of climate change, addressing some environmental issues such as water scarcity is particularly critical, especially in Chile where some misconceptions are present even in legal water management documents such as the Water Code.

This paper claims that transdisciplinary action and a co-design process allows for groundwater to be put into a broader framework. The inclusion of community leaders made the political aspects of the project evident, expanding the reflection of an initial groundwater framework in the book. At the same time, the participation of children was crucial in producing suitable material to incorporate their ongoing experiences within their communities, thus increasing public awareness of groundwater.

In terms of perception, this study confirms that public awareness of water crises is connected to the exposure of the issue. While pupils from an urban school and living in big cities such as Santiago were less aware of water issues, school children from a rural area with high/critical levels of water problems had a higher level of awareness. Nevertheless, both groups presented a low level of literacy about groundwater. Finally, this paper claims that making a book as an integrative process could be a transformative practice, not just an instrument for democratising scientific knowledge, but also for connecting people, building trust and promoting empathy among different actors, sectors and disciplines.

## Supplementary Information

ESM 1 (PDF 644 kb)
