# Supplementary material for: Improving school children’s understanding of water scarcity with a co-produced book on groundwater in Central Chile
Source: Hydrogeol J. 2023 Jun 5:1–15. Online ahead of print. doi: 10.1007/s10040-023-02641-6 (PMC10240114; doi:10.1007/s10040-023-02641-6)
Supplement: Supplementary file 1 — (PDF 644 kb) [file 10040_2023_2641_MOESM1_ESM.pdf]

Electronic Supplementary Material - Hydrogeology Journal

Improving school children's understanding of water scarcity with a co-produced book on groundwater in Central Chile

Sofía Vargas-Payera[a,b,c], Matías Taucare[b, c, d], Claudio Pareja[e], and Jessica Vejar[f]

a. ETH Zurich, D-USYS TdLab, Universitatsstrasse 16, 8092, Zurich, Switzerland.
b. Centro de Excelencia en Geotermia de Los Andes (CEGA), Facultad de Ciencias Físicas y Matemáticas, Universidad de Chile, Santiago, Chile
c. Departamento de Geología, Facultad de Ciencias Físicas y Matemáticas, Universidad de Chile, Santiago, Chile
d. Centro Avanzado para Tecnologías del Agua (CAPTA), Facultad de Ciencias Físicas y Matemáticas, Universidad de Chile, Santiago, Chile
e. Centro de Estudios del Desarrollo Regional y Políticas Públicas (CEDER), Universidad de Los Lagos, Osorno, Chile
f. Pontificia Universidad Católica de Chile, Santiago, Chile

Corresponding author: Sofía Vargas-Payera sofia.vargas@usys.ethz.ch

Table S 1: Checklist applied to the pupils' drawings and questions employed to measure their understanding of the water cycle in the first workshop during the predesign stage. (Translated from the original questionnaire in Spanish).

**Indicator**	**Yes**	**No**	**Observations**
Included Condensation			
Included Precipitation			
Included Runoff			
Included Infiltration			
Included Evaporation			
Explained orally the concept Condensation			
Explained orally the concept Precipitation			
Explained orally the concept Runoff			
Explained orally the concept Infiltration			
Explained orally the concept Evaporation			
Recognized the different uses of groundwater			
Identified domestic uses of water			
Identified industrial uses of water			
Identified other uses of water			
Explained what water scarcity is			

Table S 2 Questionnaire applied among pupils as part of measuring the book impact during the third stage (Translated from the original questionnaire in Spanish).

<table>
<tr><td colspan="2">Section 1:</td></tr>
<tr><td colspan="2">1. What elements of the book do you like the most?</td></tr>
<tr><td colspan="2">2. What was your favorite chapter of the book? Why?</td></tr>
<tr><td colspan="2">3. If you could change something about the book, what would it be?</td></tr>
<tr><td colspan="2">4. Write any comments, ideas, and recommendations that come to mind for the book</td></tr>
<tr><td>Section 2:
5. Look at the image and describe the water cycle, indicating the names of the stages</td><td>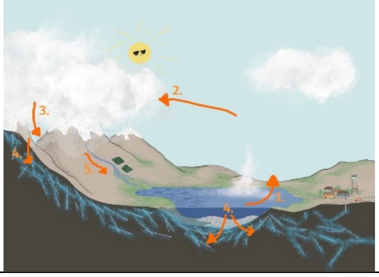</td></tr>
<tr><td colspan="2">6. Complete the following sentences about the water cycle in the box that corresponds to their number.
a. The journey of water is considered a (1) _________, that is, without beginning or end.
b. In the water cycle, this element is found in different (2) ___________, solid, liquid, and gaseous.
c. When the water evaporates, it rises to the (3) _________, where clouds form.
d. The water goes down again when the heavier clouds become (4) _________.
e. When water is in a liquid state it circulates through rivers, lakes, and seas, which is called (5) _________. In this state, water also (6) _________ underground through cracks and pores.</td></tr>
<tr><td colspan="2">Section 3:</td></tr>
<tr><td colspan="2">7. Choose if the following statements are true or false
a. Groundwater circulates through pores and cracks in rocks.
b. Under our feet is a layer of rock, and under it is a layer of water.
c. Groundwater can have different ages due to the time that it has passed since it infiltrated into the ground, until it rises to the surface again.</td></tr>
<tr><td colspan="2">8. Complete the following sentences about groundwater in the box that corresponds to their number.
a. The (1) _________ water is important because they concentrate part of the (2) _________ water that exists on our planet.
b. Groundwater moves under our feet through the (3) _________ and (4) _________ that the subsoil rocks have.
c. The (5) _________ are places in the subsoil where groundwater is most abundant.
d. For the (6) _________ of groundwater, (7) _________ are used. Through these we can obtain water for human consumption.</td></tr>
<tr><td>9. In the following image, the girl is asking her grandmother why groundwater is important and what uses it can be put to. Write in the box the answer you think Grandma will give her.</td><td>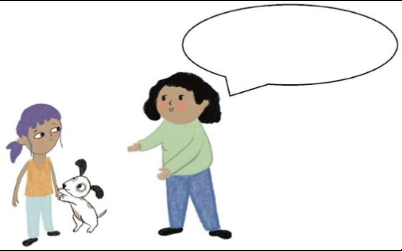</td></tr>
</table>

<table>
<tr><td colspan="2">Section 4:</td></tr>
<tr><td colspan="2">10. What is the water footprint?</td></tr>
<tr><td colspan="2">11. Name 2 examples and/or signs of water scarcity</td></tr>
<tr><td>12.In the following word search, look for the missing concepts to complete the sentences and write them in the boxes below.</td><td>C L I M Á T I C O A A
N N M N L O C A L L E
Y G G F U O D M V J S
W J Z F J B E V G I C
K Z Q E C S R Y Y B A
W W Y L K R E B M E S
M A N E J O C Q W L E
M J M R W H H X U K Z
T I E R R A O U O Í W
B L K A G U A R A M A
C V J C A L I E N T E</td></tr>
<tr><td colspan="2">a. Water is one human (1) _________.
b. The (2) _________ is the lack of rain.
c. The water (3) _________ is given both by drought and by the use and (4) _________ of water.
d. The imbalance that exists in the water that is available and that which is used is caused by two factors: one on a global scale, the (5) _________change, and the other on a (6) _________ scale, related to the management and use of water.
e. Climate change causes the earth to (7) _________ and the climate to change in all parts of the (8) _________.
f. The (9) _________ is used for most human activities and those of an economic nature require large amounts of it.
g. In Chile, at least 400,000 families depend on the water that reaches their homes via (10) _________ trucks.</td></tr>
<tr><td colspan="2">Section 5:</td></tr>
<tr><td colspan="2">13. In the fourth chapter of the book, actions against water scarcity are discussed together with Evelyn, Linda and Gloria. Next, we invite you to share with us the ideas that most caught your attention or are new to you in this chapter.</td></tr>
<tr><td>14. The girl and the dog are thinking of new ideas to deal with water scarcity. What ideas can you come up with to help them?</td><td>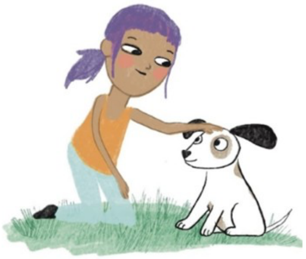</td></tr>
</table>

Table S 3 Questionnaire for school teachers, as part of measuring the book impact in the third stage (Translated from the original questionnaire in Spanish).

1. What are the strengths and weaknesses of the book in terms of its design?
2. What lessons from the current curriculum can be enhanced by working with the book?
3. What elements of the book do you find most useful both in its content and in its tools?
4. How would you use this resource in a class?
5. What improvements could the book have to be used as a pedagogical tool?
6.We invite you to comment in the following box what you thought of the book, what issues it raises and/or any other comment you may have about the book.
